# Sex, cognitive state and falls as factors associated with malnutrition: a cross-sectional study of institutionalized older adults living in a rural area of Portugal

**DOI:** 10.1186/s12889-023-15601-2

**Published:** 2023-05-02

**Authors:** Cristina Carrasco, Gorete Reis, Margarida Sim-Sim, Jose A. Parraca, Orlando Fernandes, Pablo Tomas-Carus

**Affiliations:** 1 Department of Physiology, Faculty of Medicine/Sciences, University of Extremadura, Avd. de Elvas S/N 06006, Badajoz, Spain; 2 Escola Superior de Enfermagem São João de Deus, Universidade de Évora, Évora, Portugal; 3 Comprehensive Health Research Center (CHRC), University of Évora, Évora, Portugal; 4 Departamento de Desporto E Saúde, Escola de Saúde e Desenvolvimento Humano, Universidade de Évora, Évora, Portugal

**Keywords:** Dementia, Falls, Older adults, Nursing homes, Malnutrition, Nutritional status, Portugal

## Abstract

**Background:**

Malnutrition is an underestimated geriatric problem, with a high prevalence in institutionalized older adults. The identification of risk factors for malnutrition in elderly individuals must be a priority for governmental organizations worldwide.

**Methods:**

A total of 98 institutionalized seniors were enrolled in a cross-sectional study. For the assessment of risk factors, sociodemographic characteristics and health-related information were collected. The Mini-Nutritional Assessment Short-Form test was used to assess malnutrition in the sample population.

**Results:**

A significantly greater proportion of women than men were malnourished or at risk of malnutrition. In addition, the comparative analysis revealed that comorbidity, arthritis, balance impairment, dementia and fall episodes with serious injuries were significantly more frequent in the older adults categorized as malnourished or at risk of malnutrition than in those categorized as well-nourished.

**Conclusions:**

Multivariable regression analysis revealed that being female, having a poor cognitive status and experiencing falls with injuries are the main independent factors influencing nutritional status in institutionalized older adults living in a rural area of Portugal.

## Background

While it is true that population aging is a worldwide phenomenon, a more pronounced impact has been observed in some countries and even in some regions. Within the European Union, Portugal is one of the countries with a higher proportion of older persons, and this proportion is expected to increase to 29% of the total population by 2060 [1]. For this reason, achievement of the healthy aging goal established by the World Health Organization (WHO) [2] is a major challenge that requires a multifactorial approach to formulate and implement effective policies at international, national, regional and local levels.

Among the aspects associated with increasing life expectancy, malnutrition appears to be one of the major problems faced by aging societies. According to WHO [3], malnutrition − in all its forms − includes undernutrition (wasting, stunting and underweight), inadequate intake of vitamins or minerals, overweight, obesity, and resulting diet-related noncommunicable diseases. Physiological decline inherently accompanies the aging process, affecting, among other key health indicators, nutritional status. During the aging process, there are a variety of factors influencing food intake and, consequently, nutritional status, including advancing age, female sex, declines in digestive (mastication, deglutition and gastrointestinal functions) and sensory capacities, poor oral health, chronic conditions and other disorders (loss of vision and hearing), cognitive impairment, polypharmacy and psychosocial (e.g., depression) and environmental factors (e.g., income level) [4, 5]. Given the multifactorial nature of the problem, maintenance of an adequate nutritional status can become more difficult for older adults, particularly for those living in nursing homes and other institutions [6], which is a risk factor for malnourishment. It must not be forgotten that malnutrition is ultimately going to determine activities of daily life, quality of life, dependence, risk of falling, wound healing, hospitalization and mortality, among other important aspects, in older adults [7, 8].

Currently, nutritional problems are often undetected or unaddressed in older people, despite their continuous monitoring by health care professionals [4]. In addition, there is little scientific evidence about the major risk factors for malnutrition in countries with rapidly aging populations, such as Portugal. For this reason, the aims of this study were as follows: 1) to determine a profile based on sociodemographic and health status variables of institutionalized older adults according to their nutritional status and 2) to evaluate the involvement of these variables as risk factors for malnutrition.

## Methods

### Participants

The minimum representative sample size of the total population of older people ≥ 65 years old (*n* = 182,988) was calculated using the epidemiologic statistics software OpenEpi [9], taking into account an estimated prevalence of undernutrition of 50.0% [10]. The parameters under consideration were as follows: statistical power of 80.0%, alpha = 0.5 and effect size 1, as previously described by Santos et al. [10]. Thus, a sample size of 125 older adults was obtained. Participants were seniors who were institutionalized (day center/nursing home) in the homes run by the National Confederation of the Solidarity Institutions in the various municipalities of the district of Évora (Portugal). The inclusion criteria were as follows: i) males and females aged ≥ 65 years old; ii) day care center users or nursing home residents; and iii) the ability to speak and understand Portuguese. The data collection was performed from March 2018 to June 2018. Participants with an incomplete assessment of malnutrition were excluded from the data analysis. Thus, a total of 98 older adults (32 males and 66 females) were finally included in the cross-sectional study.

All older adults gave full informed written consent for participation. The University of Évora Ethics Committee for research in the areas of human health and well-being (reference number 16–012) approved this study, which followed the updates of the Declaration of Helsinki.

### Measures, design and procedures

The variables of interest were collected by trained project assistants through computer-assisted face-to-face structured interviews with the participants; then, anthropometric measurements were performed. The researchers also encouraged caregivers (family members and nursing home staff) of more vulnerable seniors to collaborate by answering some questions, especially those related to falls and associated injuries.

#### Sociodemographic characteristics and health-related information

Sociodemographic characteristics were recorded and categorized as described below: sex (female/male), age (years), retirement age (years), civil status (married/unmarried/widower/separated-divorced/unmarried partner), form of institutionalization (day care center/nursing home), living status (alone/spouse/family/children/others), education level (< high school (≤ 12 years) /high school or higher (> 12 years)) and income level (do not know-do not answer/ < 350€/350–550€/550–750€).

Furthermore, the participants´ self-reported health status was assessed by recording the following variables: medical histories (chronic diseases and other disorders), current medication (number and types of drugs) and fall events (within the past 6 and 12 months, as well as fall-related injuries within the past year).

#### Nutritional status

To identify older adults who were malnourished or at risk of malnutrition, the Mini-Nutritional Assessment Short-Form (MNA-SF) test [11] was used. This validated screening tool is a six-item questionnaire on food intake, weight loss, mobility, psychological stress or acute disease, presence of dementia or depression, and body mass index (BMI) or calf circumference. The total test score ranges from 0–14 points: scores of 12–14 indicate a normal nutritional status; scores of 8–11 indicate a risk of malnutrition; and scores of 0–7 indicate malnutrition. According to the individual MNA-SF total score, the research team categorized the participants into two subgroups: 1) malnourished or at risk of malnutrition (M/RM), for scores equal or below 11; and 2) well-nourished (WN) for scores 12 and above.

#### Body composition

The body weight (kg) and height (m) were measured using a digital balance (Seca 760, Hamburg, Germany) and a stadiometer (Seca 206, Hamburg, Germany); then, BMI was calculated (kg/m^2^), and participants were categorized according to their values (< 25/ > 25). Waist and thigh perimeters were measured using anthropometric tape as described elsewhere [12]. Other interesting variables related to body composition were assessed by the trained project assistants using a clinically validated body composition monitor by the bioelectrical impedance method (OMRON HBF-306C, Kyoto, Japan), including body fat (%), muscle mass (kg), visceral fat level and basal metabolic rate (kcal).

#### Sleep

To assess the sleep of the participants, the research team recorded the number of hours of sleep. Additionally, daytime sleepiness was measured using the Portuguese version [13] (Cronbach's alpha = 0.75) of the Epworth Sleepiness Scale (ESS) [14]. The ESS is an 8-item questionnaire on the usual chance of dozing off or falling asleep while engaged in eight different activities, rating on a 4-point scale (0–3) by the respondent. The total score is the sum of the 8 item scores and ranges from 0 to 24. According to the individual ESS total score, participants were categorized into two subgroups: normal daytime sleepiness (0–10) and excessive daytime sleepiness (11–24).

#### Cognitive mental state

The Portuguese version [15] (Cronbach's alpha = 0.46) of the Mini-Mental State Examination (MMSE) [16] was used to assess the cognitive state of the study participants. The 11 items of the MMSE include tests of orientation, registration, recall, calculation and attention, naming, repetition, comprehension, reading, writing and drawing. Maximum total score is 30. The categorized subgroups were as follows: dementia (9–11), cognitive impairment (12–24), suspected pathology (< 27) and normal cognition (≥ 27).

#### Fear of falling

The Fall Efficacy Scale-International [17] (Portuguese version [18]: interrater reliability of *r* = 0.62; interclass correlation coefficient (ICC = 0.859) was used to measure the level of concern about falling during 16 daily tasks related to social and physical activities, both inside and outside home. Each task is rated on a four-point Likert-type scale (1 = “not at all concerned” to 4 = “very concerned”); the total score ranges from 16—64. Participants were categorized into three subgroups: low concern (16–19), moderate concern (20–27) and high concern (28–64).

#### Degree of dependence

To measure the functional independence of participants in performing activities of daily living (clothing, nourishment, personal hygiene and transfers), the Barthel Scale [19] was used (Portuguese version [20]: Cronbach's alpha = 0.96). In this 10-item questionnaire, each item receives a score of 0, 1, 2 or 3, reaching a total of 20 for individuals who are independent. According to their total scores, the study participants were categorized into two subgroups: moderate dependence (13–19) and independence (20).

### Statistical analysis

Values are expressed descriptively in terms of numbers of cases, mean ± standard deviation (SD) (continuous variables) and frequency (%) (categorical variables). Continuous data were assessed for normality using the Kolmogorov–Smirnov test. Significant differences between subgroups (M/RM/WN) were analyzed using unpaired t tests and chi-square tests for continuous and categorical variables, respectively. Then, multivariable logistic regression analysis was applied to detect significant associations between the dependent variable (nutritional status) and the independent variables that were significant (*p* < 0.05) in the univariate analyses, including sex, comorbidity, arthritis, balance impairment, cognitive state and falls with injuries. For this purpose, the regression model was built using the Enter Method. In addition, since risk factors for malnourishment must be easily and quickly interpreted, all categorical variables were dichotomized. The goodness-of-fit of the model was assessed with the Homer-Lemeshow test. The results are presented as adjusted odds ratios (AORs) with 95% confidence intervals (CIs). Statistical analyses were carried out using IBM® SPSS® Statistics (v.25, IBM, New York, USA) for Windows. Statistical significance was established at *p* < 0.05.

## Results

As shown in Table 1, a total of 98 older adults (women, 67.3%; men, 32.7%) were included in the analysis. The mean age was 84.1 ± 6.7 years, and the mean retirement age was 59.4 ± 8.4 years. Most participants were widowers (63.3%) and lived alone (55.1%). With respect to the form of institutionalization, the percentages of day care center users and nursing home residents were 46.9% and 53.1%, respectively. All of them (100%) had an educational level equal to or lower than compulsory education (≤ 12 years). The monthly income was in the range of 550—750€ only for 9.2% of the older adults analyzed. In addition, the statistical comparison of the M/RM and WN subgroups is shown in Table 1. MNA-SF results revealed that 22.4% of study participants were categorized as malnourished (2.0%) or at risk of malnutrition (20.4%) (data not shown), and 77.6% were categorized as well nourished. A significantly greater proportion of women belonged to the M/RM subgroup (90.9% vs. 60.5%; *p* = 0.007), unlike men (9.1% vs. 39.5%, *p* = 0.007). Regarding civil status and nutritional status, an association of borderline statistical significance was observed; in this sense, it is remarkable that the number of older adults who had a spouse or an unmarried partner was higher in the WN subgroup than in the M/RM subgroup. Moreover, the results showed that income level could influence the risk of malnutrition. However, the high proportion of participants (21.4%) who did not answer this question does not allow conclusions to be drawn. For this reason, the research team decided not to include this independent variable in the multivariable logistic regression model, despite the significant differences between subgroups.

**Table 1:** General characteristics of the study population (*n* = 98) according to the nutritional status

			**Nutritional status**		
		**Total**	**M/RM**	**WN**	***P*-value**^**d**^
	**Category**	**100.0% (98)**	**22.4% (22)**	**77.6% (76)**	
Sex ^a^	Female	67.3 (66)	90.9 (20) ^c^	60.5 (46)	0.007
	Male	32.7 (32)	9.1 (2) ^c^	39.5 (30)	
Age, years ^b^	-	84.1 ± 6.7	82.2 ± 6.3	84.7 ± 6.7	0.120
Retirement age, years ^b^	-	59.4 ± 8.4	57.5 ± 9.2	60.0 ± 8.1	0.220
Civil status ^a^	Married	22.4 (22)	13.6 (3)	25.0 (19)	0.064
	Unmarried	9.2 (9)	9.1 (2)	9.2 (7)	
	Widower Separated/divorced	63.3 (62)	68.2 (15)	61.8 (47)	
	Unmarried partner	2.0 (2)	9.1 (2)	-	
		3.1 (3)	-	3.9 (3)	
Institutionalization ^a^	Day care centre	46.9 (46)	50.0 (11)	46.1 (35)	0.744
	Nursing home	53.1 (52)	50.0 (11)	53.9 (41)	
Living with ^a^	Alone	55.1 (54)	50.0 (11)	56.6 (43)	0.682
	Spouse	7.1 (7)	4.5 (1)	7.9 (6)	
	Family	2.0 (2)	-	2.6 (2)	
	Children	11.2 (11)	18.2 (4)	9.2 (7)	
	Others	24.5 (24)	27.3 (6)	23.7 (18)	
Education level ^a^	< High school	100.0 (98)	100.0 (22)	100.0 (76)	-
	High school or higher	-	-	-	
Income level ^a^	Dk/Da	21.4 (21)	40.9 (9) ^c^	15.8 (12)	0.022
	< 350€	36.7 (36)	40.9 (9)	35.5 (27)	
	350–550€	32.7 (32)	18.2 (4)	36.8 (28)	
	550–750€	9.2 (9)	-	11.8 (9)	

^a^Values expressed as % (n). *p-*values of Chi-square analysis

^b^Values expressed as the mean ± standard deviation. *p-*values of t test analysis*. M/RM* malnourished or at risk of malnutrition*, WN* well-nourished*, Dk* Do not know*, Da* Do not answer. *p-*value < 0.05

^c^vs. WN subgroup

^d^*p-*value of χ2 test or t test

On the other hand, variables related to the health status of the participants are descriptively expressed in Table 2. A total of 53.7% of individuals were diagnosed with 3 or more conditions. Poliomyelitis (25.3%), diabetes (17.9%), cardiovascular (hypertension: 51.6%), osteoarticular (arthrosis: 32.6%), and neurologic (depression: 14.7%) diseases were the most prevalent clinical conditions in the study population. Other disorders affecting more than half of the older adults were nocturia (65.2%), balance (58.4%) and dizziness (52.8%). Polypharmacy was only reported for 16.3% of the participants; most of them (62.22%) declared that they were taking medication other than psychotropic/anxiolytic drugs alone (3.1%) or in combination with anti-inflammatory or diuretic drugs (3.1%). Regarding body composition, a total of 77.3% of participants had BMI values > 25 (overweight or obese). Other interesting findings in the study population were normal daytime sleepiness (94.6%) and the presence of cognitive impairment (54.7%); furthermore, it should be mentioned that 40.0% of seniors experienced falls within the past year, edema was the most common fall-related injury (14.9%), and the percentage of totally dependent individuals was 64.3% when the study was carried out. The comparative analysis between subgroups revealed a significantly higher proportion of older adults, with two conditions diagnosed in the M/RM subgroup (28.6% vs. 10.8%; *p* = 0.043); in addition, arthritis (19.0% vs. 4.1%; *p* = 0.020) and balance impairment (80.0% vs. 52.2%, *p* = 0.026) were more prevalent in these individuals than in WN individuals. From a statistical point of view, no significant differences were observed either in BMI or other variables of body composition; however, a higher proportion of older adults in the WN subgroup were overweight or obese compared to the M/RM subgroup (81.3% vs. 63.6%; *p* = 0.081). Regarding MMSE score, the number of malnourished or at risk of malnutrition participants included in the category of dementia was significantly higher than the number of well-nourished participants (18.2% vs. 2.7%; *p* = 0.035). Likewise, the M/RM subgroup experienced more falls over the last year (6 months: 40.9% vs. 20.5%; *p* = 0.054/12 months: 50.0% vs. 37.0%; *p* = 0.275) and suffered more serious injuries (45.5% vs. 15.1%; *p* = 0.003), such as edema (13.6% vs. 2.6%; *p* = 0.045), than the WN subgroup; consequently, their fear of falling was also higher (high concern: 54.5% vs. 32.9%; *p* = 0.188). Finally, it is noteworthy that there was a high proportion of older adults with moderate dependence who were malnourished or at risk of malnutrition (81.8% vs. 59.2%; *p* = 0.148).

**Table 2:** Health status of the study population (*n* = 98) according to the nutritional status

			Nutritional status		
		Total	M/RM	WN	*P*-value^**d**^
Variable	Category	100.0% (98)	22.4% (22)	77.6% (76)	
Diagnosed conditions					
Comorbidity ^a^	None	20.0 (19)	14.3 (3)	21.6 (16)	0.043
	1 condition	11.6 (11)	-	14.9 (11)	
	2 conditions	14.3 (14)	28.6 (6) ^c^	10.8 (8)	
	3 or more conditions	53.7 (51)	57.1 (12)	52.7 (39)	
Chronic diseases ^a^	Hypertension	51.6 (49)	42.9 (9)	54.1 (40)	0.365
	Cardiovascular	32.6 (31)	47.6 (10)	28.4 (21)	0.097
	Vascular	32.6 (31)	33.3 (7)	32.4 (24)	0.938
	Arthrosis	32.6 (31)	28.6 (6)	33.8 (25)	0.653
	Poliomyelitis	25.3 (24)	28.6 (6)	24.3 (18)	0.693
	Osteoporosis	17.9 (17)	14.3 (3)	18.9 (14)	0.625
	Diabetes	17.9 (17)	23.8 (5)	16.2 (12)	0.423
	Depression	14.7 (14)	9.5 (2)	16.2 (12)	0.445
	Lung	12.6 (12)	19.0 (4)	10.8 (8)	0.316
	Ménière´s syndrome	8.4 (8)	14.3 (3)	6.8 (5)	0.273
	Arthritis	7.4 (7)	19.0 (4) ^c^	4.1 (3)	0.020
	Cancer	6.3 (6)	14.3 (3)	4.1 (3)	0.089
	Other osteoarticular	5.3 (5)	9.5 (2)	4.1 (3)	0.322
	Parkinson´s disease	2.1 (2)	-	2.7 (2)	0.446
	Epilepsy	1.1 (1)	-	4.8 (1)	0.059
Other disorders ^a^	Nocturia	65.2 (58)	75.0 (15)	62.3 (43)	0.295
	Balance	58.4 (52)	80.0 (16) ^c^	52.2 (36)	0.026
	Dizziness	52.8 (47)	65.0 (13)	49.3 (34)	0.215
	Vision	43.8 (39)	50.0 (10)	42.0 (29)	0.527
	Feet	38.2 (34)	50.0 (10)	34.8 (24)	0.217
	Audition	38.2 (34)	30.0 (6)	40.6 (28)	0.391
	Urinary incontinence	23.9 (21)	35.0 (7)	20.6 (14)	0.184
Medication					
Polypharmacy (≥ 5 drugs) ^a^	Dk/Da	30.6 (30)	45.5 (10)	26.3 (20)	0.228
	No	53.1 (52)	40.9 (9)	56.6 (43)	
	Yes	16.3 (16)	13.6.0 (3)	17.1 (13)	
Drugs ^a^	Dk/Da	31.6 (31)	45.5 (10)	27.6 (21)	0.333
	Psychotropic/ anxiolytic	3.1 (3)	4.5 (1)	2.6 (2)	
	Mixture ^e^	3.1 (3)	4.5 (1)	2.6 (2)	
	Other drugs	62.2 (61)	45.5 (10)	67.1 (51)	
Body composition/metabolism					
BMI ^a^	< 25	22.7 (22)	36.4 (8)	18.7 (14)	0.081
	> 25	77.3 (75)	63.6 (14)	81.3 (61)	
Waist perimeter (cm) ^b^	-	99.98 ± 10.59	97.08 ± 13.04	100.83 ± 9.70	0.145
Thigh perimeter (cm) ^b^	-	105.74 ± 10.04	106.55 ± 12.61	105.50 ± 9.25	0.719
Body fat (%) ^b^	-	34.20 ± 11.77	35.59 ± 10.46	33.93 ± 11.34	0.702
Muscle mass (kg) ^b^	-	27.17 ± 5.90	25.26 ± 6.51	27.53 ± 5.78	0.292
Visceral fat level ^b^	-	12.71 ± 4.41	11.00 ± 3.91	13.04 ± 4.46	0.205
Basal metabolic rate (kcal) ^b^	-	1412.59 ± 151.71	1386.67 ± 183.99	1417.55 ± 146.54	0.580
Sleep					
Sleeping hours ^a^	Dk/Da	37.8 (37)	27.3 (6)	40.8 (31)	0.347
	< 8	11.2 (11)	18.2 (4)	9.2 (7)	
	> 8	51.0 (50)	54.5 (12)	50.0 (38)	
Daytime sleepiness ^a^	Normal	94.6 (88)	95.5 (21)	94.4 (67)	0.843
	Excessive	5.4 (5)	4.5 (1)	5.6 (4)	
Cognitive state ^a^					
	Dementia	6.3 (6)	18.2 (4) ^c^	2.7 (2)	0.035
	Cognitive impairment	54.7 (52)	59.1 (13)	53.4 (39)	
	Suspected pathology	20.0 (19)	9.1 (2)	23.3 (17)	
	Normal cognition	18.9 (18)	13.6 (3)	20.5 (15)	
Falls					
6 months ^a^	-	25.3 (24)	40.9 (9) ^c^	20.5 (15)	0.054
12 months ^a^	-	40.0 (38)	50.0 (11)	37.0 (27)	0.275
Falls with injuries ^a^	-	22.1 (21)	45.5 (10) ^c^	15.1 (11)	0.003
Types of injuries ^a^	Minor				
	Edema	14.9 (14)	18.2 (4)	13.7 (10)	0.603
	Excoriation	9.6 (9)	18.2 (4)	6.8 (5)	0.112
	Others	1.1 (1)	4.5 (1)	4.1 (3)	0.929
	Serious				
	Edema	5.3 (5)	13.6 (3) ^c^	2.7 (2)	0.045
	Fracture	5.3 (5)	4.5 (1)	5.5 (4)	0.863
	Abrasion	1.1 (1)	-	1.4 (1)	0.581
	Distention	1.1 (1)	4.5 (1)	-	0.067
Fear of falling ^a^	Low concern	25.0 (23)	18.2 (4)	27.1 (19)	0.188
	Moderate concern	37.0 (34)	27.3 (6)	40.0 (28)	
	High concern	38.0 (35)	54.5 (12)	32.9 (23)	
Degree of dependence					
	Dk/Da	10.2 (10)	4.5 (1)	11.8 (9)	0.148
	Moderate dependence	64.3 (63)	81.8 (18)	59.2 (45)	
	Independence	25.5 (25)	13.6 (3)	28.9 (22)	

^a^Values expressed as % (n); *p*-values of Chi-square analysis

^b^Values expressed as the mean ± standard deviation. *p*-values of t test analysis. *M/RM* malnourished or at risk of malnutrition, *WN* well-nourished, *Dk/Da* Do not know / Do not answer. *p*-value < 0.05

^c^vs. WN subgroup

^d^*p-*value of χ2 test or t test

^e^(psychotropic/anxiolytic + anti-inflammatory or diuretics drugs)

Factors associated with malnutrition were analyzed in depth using a multivariable logistic regression model (Table 3). The analysis showed that being a woman (AOR 0.098, 95% CI 0.010–0.990) increased the risk of malnutrition in the studied older Alentejo population. In addition, an association of borderline statistical significance between low cognitive status (dementia) and the risk of being malnourished was noted (AOR 0.059, 95% CI 0.003–1.001). On the other hand, older adults who experienced falls with injuries within the past year were more likely to be malnourished (AOR 12.102, 95% CI 2.545–57.537) in relation with those with no falls.

**Table 3:** Multiple regression analysis of associated factors for nutritional status in the study population (*n* = 98)

Variable	Category	Adjusted OR (95%CI)
Sex	Male	-
	Female	0.098 (0.010–0.990)
Diagnose conditions		
Comorbidity	Yes	-
	No	3.190 (0.474–21.492)
Chronic diseases—Arthritis	Yes	-
	No	0.655 (0.073–5.850)
Other disorders—Balance	Yes	-
	No	4.376 (0.826–23.183)
Cognitive state	Normal	-
	Dementia	0.059 (0.003–1.001)
	Cognitive impairment	0.470 (0.077–2.886)
	Suspected pathology	6.608 (0.376–116.053)
Falls with injuries	Yes	-
	No	12.102 (2.545–57.537)

Reference categories (-). *OR* Odds Ratios; *CI 95%* Confidence interval

## Discussion

Malnutrition appears to be a prevalent problem in the elderly population, especially in older adults living in nursing homes, where the reported prevalence among residents is between 15 and 40%; for that reason, it is also referred to as a geriatric syndrome [21, 22]. In general terms, the multiple factors influencing the risk of malnourishment in elderly individuals can be categorized into physical, psychological, social, oral health-related and eating-related factors [4]; consequently, the simultaneous occurrence of several risk factors is expected to aggravate the risk for malnutrition [23].

In comparison with other studies analyzing nutritional status in institutionalized older adults [6, 21, 23–25], our results showed a lower proportion of older adults who were malnourished or at risk of being so, similar to those observed by Bakker et al. [8]. Previous literature has reported an association between advanced age and malnutrition [23, 24]. However, this factor does not seem to be determinant among the Portuguese older population, as shown by this and other studies performed in community-dwelling older adults [10]. Furthermore, currently available data point out that other sociodemographic variables are significantly related to a poor nutritional status regardless of where institutionalized older adults live, including sex [5, 26], widowhood [5, 10], low educational status [8, 24], insufficient income and duration of the stay in the nursing home [24]. In this line, our findings revealed that members of the M/RM group were mostly women, unpartnered, undereducated and with low income; this risk profile is also shared in noninstitutionalized older adults living in both Portugal [10] and neighboring Spain [27] due to the similar sociodemographic characteristics of their older populations.

With regard to health status, our results showed that malnutrition was significantly associated with comorbidity (only for two conditions diagnosed), arthritis and balance impairment but not with polypharmacy and other prevalent chronic conditions, such as cardiovascular diseases and diabetes. Other researchers have reported mixed results. A Syrian study [24] observed that the nutritional status of nursing home residents was significantly affected by the number of chronic diseases and, consequently, by the number of medicines taken. However, comorbidity and polypharmacy were not reported as risk factors in another study carried out in Germany [23]. Furthermore, many other health-related factors, such BMI, impaired cognitive function and disability, are associated with an increasing risk of becoming malnourished in institutionalized elderly people [21]. In the present study, the M/RM subgroup had lower BMI, a significantly worse cognitive state and a higher proportion of individuals moderately dependent on their caregivers for daily life activities. In this sense, it is well known that as dementia, dependence and care needs increase, so does the risk of malnourishment [22, 28]. However, this relationship can work in both ways and has multiple causes that have been largely described [5, 29]. As Malara et al. [30] point out, dementia is itself a risk factor for malnutrition, especially in later stages of the disease when several physiological dysfunctions occur, such as dysphagia. Consequently, neurological disorders (e.g., dementia and Parkinson's disease) and disability are more common in malnourished older adults than in well-nourished adults [21, 26]. These findings suggest that this is a vicious cycle leading to sarcopenia, functional and balance impairments and an increasing risk of frailty in institutionalized older adults [31], with a high impact on performance of daily life activities and therefore autonomy [30]. As in other studies [5, 21, 26, 29, 30], our results from regression analysis pointed out the association between dementia and the risk of being malnourished. However, this finding was of borderline statistical significance, likely due to the underrepresentation of subjects with low cognitive status in the studied population. Finally, it should not be forgotten that all the above-mentioned health issues influence the risk of falling in the older population, as explained elsewhere [32]. A previous study observed that falls are significantly associated with a high probability of being malnourished or at risk of malnourishment in institutionalized older adults [26], in the line with our findings derived from the logistic regression analysis.

To the best of our knowledge, this is one of the few studies analyzing the profile and risk factors for malnutrition in Portuguese institutionalized older adults living in rural areas. As with many other public health problems in elderly individuals, malnutrition is influenced by the intrinsic characteristics of each nation, including sociodemographic and cultural factors, leading to inconsistent results across studies. Thus, in Mediterranean countries such as Portugal, it is expected that a very aged population with different risk factors from those identified in other populations of the world will be found. In contrast with other research, the present study has focused on an institutionalized older population of a rural area, without excluding participants with varying degrees of cognitive impairment. Therefore, further studies are needed to identify these differences at international, national and regional levels, taking into account all the diverse realities of this life stage.

Some potential limitations need to be addressed. First, the cross-sectional design allows an initial approach to profiling risks before performing longitudinal studies in countries/regions that have not been previously analyzed; however, the causal relationships between the factors identified and malnutrition cannot be established in this type of study. Second, the risk of sample bias must also be considered due to a) the sample size, b) the overrepresentation of female participants in the studied population, c) the inclusion of both day care center users and nursing home residents, and d) the unequal sample sizes of subgroups; on balance, this could have an influence on the detection of significant associations. Third, the method of data collection based on self-reported data and retrospective recall makes that some of the studied variables may be underestimated, and recall bias must be considered, despite the face-to-face interviews being conducted by researchers with the help of caregivers. Finally, data analysis was performed by a blinded outcome assessor to reduce the possibility of bias, but the effect of other factors that were not investigated, as well as interaction effects and residual confounding, cannot be ruled out.

In summary, our study confirms previous findings about the multifactorial nature of malnutrition in the aged population. This evidence should be taken into account to integrate new knowledge into clinical practice. As Bauer et al. [22] point out, it is essential to promote independence in nursing home residents and help them gain individual resources. In this sense, innovative initiatives in institutionalization have been launched to promote autonomy in the frailest seniors. A good example of this is the shared-housing arrangements, where cohabitation and daily life routines are organized from a different point of view compared to nursing homes. Recently, Meyer et al. [29] observed that the prevalence of malnutrition in shared-housing arrangements was lower than that in nursing homes and similar to that reported by community-dwelling seniors. For authors, the maintenance of family-like structures, the performance of activities of daily living (e.g., food shopping and cooking), and the creation of an adequate environment (in terms of noise, lighting, routines, etc.) promote the autonomy of persons with dementia, which is reflected in their nutritional status. This approach is shared by other researchers, who claim for a more proactive and social life as a solution for malnutrition in institutionalized older adults. For them, some improving strategies in daily practice, such as mealtime interventions (flexible schedules, availability of snacks, etc.) and adaptations in staffing planning, may ensure adequate nutrient intake in institutionalized older adults, especially for the oldest and most frail [28, 32]. On the other hand, the current lack of agreed-upon diagnostic criteria and a gold-standard test, among other aspects, makes it difficult to build a social awareness of the magnitude of malnutrition in older people [21, 22]. Therefore, identification of the risk factors for this public health issue must be a priority for governmental organizations worldwide, without forgetting the influence of sex on diverse aspects of life, including health-related problems. For this reason, we believe it is essential to carry out this kind of study from a gender perspective.

## Conclusions

The findings revealed that the prevalence of malnutrition was lower than expected in institutionalized older adults living in a rural area of Portugal. A thorough analysis of the results showed that being female, having a poor cognitive status and experiencing falls with injuries are the main independent factors influencing nutritional status in the studied population. As with many other public health problems in elderly individuals, malnutrition is determined by the intrinsic characteristics of each nation, including sociodemographic and cultural aspects. Since malnourishment is currently underestimated, it is of vital importance to identify risk factors to define and implement protocols for screening and monitoring nutritional status.

## Data Availability

The datasets used and/or analyzed during the current study are available from the corresponding author upon reasonable request.

## Abbreviations

MNA-SF: Mini-Nutritional Assessment Short-Form
BMI: Body mass index
M/RM: Malnourished or at risk of malnutrition subgroup
WN: Well-nourished subgroup
ESS: Epworth Sleepiness Scale
MMSE: Mini-Mental State Examination
ICC: Interclass correlation coefficient
SD: Standard deviation
AORs: Adjusted odds ratios
CIs: Confidence intervals
SPSS: Statistical Package for Social Sciences
